# Solution structure of the cytochrome P450 reductase–cytochrome *c* complex determined by neutron scattering

**DOI:** 10.1074/jbc.RA118.001941

**Published:** 2018-02-23

**Authors:** Samuel L. Freeman, Anne Martel, Juliette M. Devos, Jaswir Basran, Emma L. Raven, Gordon C. K. Roberts

**Affiliations:** From the Departments of ‡ Chemistry and; ‖ Molecular and Cell Biology and; ¶ Leicester Institute of Structural and Chemical Biology, University of Leicester, Leicester LE1 7RH, United Kingdom and; § Institut Laue-Langevin, 71 avenue des Martyrs, 38042 Grenoble, France

**Keywords:** neutron scattering, protein-protein interaction, cytochrome P450, cytochrome c, electron transfer, protein structure, cytochrome P450 reductase, SANS, transient complex, solution structure, protein structural dynamics

## Abstract

Electron transfer in all living organisms critically relies on formation of complexes between the proteins involved. The function of these complexes requires specificity of the interaction to allow for selective electron transfer but also a fast turnover of the complex, and they are therefore often transient in nature, making them challenging to study. Here, using small-angle neutron scattering with contrast matching with deuterated protein, we report the solution structure of the electron transfer complex between cytochrome P450 reductase (CPR) and its electron transfer partner cytochrome *c*. This is the first reported solution structure of a complex between CPR and an electron transfer partner. The structure shows that the interprotein interface includes residues from both the FMN- and FAD-binding domains of CPR. In addition, the FMN is close to the heme of cytochrome *c* but distant from the FAD, indicating that domain movement is required between the electron transfer steps in the catalytic cycle of CPR. In summary, our results reveal key details of the CPR catalytic mechanism, including interactions of two domains of the reductase with cytochrome *c* and motions of these domains relative to one another. These findings shed light on interprotein electron transfer in this system and illustrate a powerful approach for studying solution structures of protein–protein complexes.

## Introduction

The complexes formed by proteins involved in electron transfer are often relatively weak and transient (1, 2). This generally makes the complexes difficult to crystallize, although some such transient complexes have been successfully studied by NMR techniques (3–8).

Cytochrome P450 reductase (CPR)^3^ (9–12) has an important physiological role as a key component of the P450 mono-oxygenase system of the endoplasmic reticulum, which plays a central role in drug metabolism (13). Cytochromes P450 catalyze the insertion of one atom of molecular oxygen into their substrates with the reduction of the other atom to water, a reaction requiring two electrons that, in the case of the drug-metabolizing P450s, are supplied by CPR (11, 14). In the liver endoplasmic reticulum, cytochrome P450s are present in excess over CPR, with a molar ratio of cytochrome P450/CPR as high as 20:1, so that electron transfer must occur in transient complexes whose lifetime has been estimated at ∼200 ms (15). CPR accepts electrons from the obligatory two-electron donor NADPH onto its FAD cofactor and transfers them via its FMN cofactor to a wide range of different P450s; the two electrons are donated one at a time at two distinct steps in the cytochrome P450 reaction cycle (16, 17), and it is possible that the complex dissociates between these two electron transfer steps (15).

CPR, like other members of the family of diflavin reductases (18–20), has three domains: an FMN-binding domain, an FAD- and NADPH-binding domain, and a “linker” domain; the FMN domain is connected to the linker and FAD domains through a highly flexible “hinge.” The compact conformation of truncated soluble CPR seen in the X-ray crystal structure (10, 12) is well suited for electron transfer from FAD to FMN as the two isoalloxazine rings are less than 4 Å apart. However, in this conformation, it is difficult to see how an electron transfer partner protein could approach close enough to the FMN for electron transfer to occur, and there is good evidence that the domains of CPR must move relative to one another to allow access of the redox partners to the FMN cofactor (9, 11, 21–28).

CPR was first isolated and identified as an NADPH-dependent cytochrome *c* reductase (29); although cytochrome *c* (cyt *c*) is unlikely to be a physiological redox partner of CPR, the electron transfer reaction between CPR and cyt *c* is widely used as a standard model to characterize the activity of CPR, and it is probable that the binding sites on CPR for cyt *c* and cytochrome P450 are at least substantially overlapping (30, 31). We have now used the powerful combination of small-angle neutron scattering (SANS) with contrast variation using deuterated protein to determine a low-resolution solution structure of the complex between cytochrome *c* and CPR lacking only the N-terminal membrane-binding anchor.

## Results

### Characterization of CPR K75E/R78E/R108Q

To facilitate the study of the CPR–cyt *c* complex, we used the K75E/R78E/R108Q mutant of CPR in which two salt bridges stabilizing the compact conformation (see below) are abolished. This mutant shows a 5-fold decrease in the *K_m_* for cyt *c* and a doubling of the rate constant *k*_cat_ for cyt *c* reduction (27). There is also evidence from SAXS for a more extended conformation of the oxidized form of the mutant relative to the wildtype, although the partial reduction of the flavins by X-ray–induced photoelectrons (27) complicates the interpretation of these experiments.

**Figure 1.**
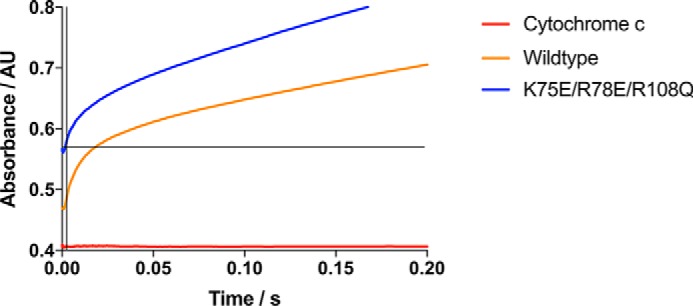
**Burst kinetics of reduction of cytochrome *c* on rapid mixing with NADPH-reduced CPR.** Wildtype CPR (*orange*) or K75E/R78E/R108Q mutant CPR (*blue*) was mixed with cytochrome *c* in a stopped-flow spectrophotometer under anaerobic conditions. The baseline on mixing cytochrome *c* with buffer is shown in *red. AU*, absorbance units.

Rapid mixing of prereduced CPR and cyt *c* leads to a burst of cyt *c* reduction, essentially within the experimental dead time of the stopped-flow spectrophotometer, by those CPR molecules that are in a reactive (“open”) state followed by a slower reduction of cyt *c* by those CPR molecules that exist in a cyt *c*–unreactive (“closed”) conformation (32). Fig. 1 shows the kinetic traces of cyt *c* reduction following rapid mixing of prereduced CPR and cyt *c*. For the wildtype enzyme, ∼20% reduction of cyt *c* takes place within the 2-ms dead time of the instrument in reasonable agreement with the report by Haque *et al.* (32) of 33% reduction within 4 ms. For the K75E/R78E/R108Q mutant, however, a much higher proportion of the reduction takes place in the dead time, suggesting that in the reduced state the mutant is predominantly (>80%) in the reactive conformation.

To establish whether the mutant also has an increased proportion of the extended conformation in the oxidized state, we carried out solution scattering studies of the free wildtype and mutant CPR using SANS. The hydrodynamic parameters for wildtype and mutant CPR, derived from the scattering curves in Fig. S1, are given in Table 1. It can be seen that the *R_g_* and *D*_max_ values for the mutant are greater than those for the wildtype.

**Table 1:** **Hydrodynamic parameters for wildtype and mutant CPR**

Sample	*R_g_*	*D*_max_	Model*^a^*					
			Crystal structure + Huang *et al.* model			Crystal structure + ΔTGEE mutant model		
			*f*_compact_*^b^*	*f*_extended_*^c^*	χ^2^	*f*_compact_*^b^*	*f*_extended_*^c^*	χ^2^
	Å	Å						
Wildtype	24.7 ± 0.1	71	0.90	0.10	1.64	0.90	0.10	1.75
K75E/R78E/R108Q mutant	25.5 ± 0.2	73	0.84	0.16	1.82	0.67	0.33	1.20

*^a^* The models used to analyze the scattering data in terms of a two-state equilibrium are described in the text. In both cases, the compact state is described by the crystal structure of oxidized CPR; the extended structure is described *either* by the model of Huang *et al.* (27) *or* by the structure of the ΔTGEE mutant (24). The goodness of fit to the scattering curve is given by the χ^2^ statistic.

*^b^* Fraction of the compact conformation.

*^c^* Fraction of the extended conformation.

**Figure 2.**
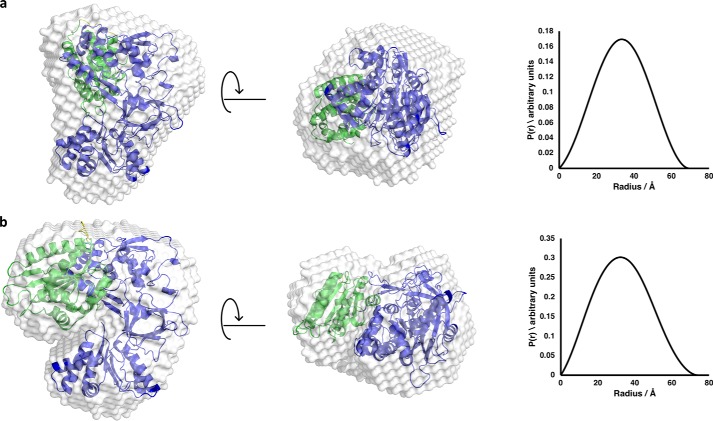
**Scattering envelopes of wildtype CPR and K75E/R78E/R108Q mutant CPR with atomic models superimposed together with the *P*(*r*) plots derived from the scattering data (*right*).** In *a* (wildtype CPR), the model is the crystal structure of wildtype CPR (12). In *b* (mutant CPR), the model is one obtained from the wildtype crystal structure after a conformational search allowing motion of the residues in the flexible hinge as described under “Experimental procedures”. In both cases, the envelope and structure on the *right* are related to those on the *left* by a 90° rotation in the direction shown by the *arrows*. In the structural models superimposed on the scattering envelopes, the FAD and linker domains are *blue*, and the FMN domain is *green*.

Fig. 2 shows average scattering envelopes for the two proteins constructed *ab initio* using DAMMIF together with the respective intraparticle pairwise distance distribution (*P*(*r*)) plots; *P*(*r*) plots are the real space representation of the scattering data, obtained by carrying out an indirect Fourier transform on the reciprocal space data. These data clearly indicate a slightly more extended average structure for the mutant than for the wildtype protein in the oxidized state with a visible “cleft” in the mutant structure.

**Figure 3.**
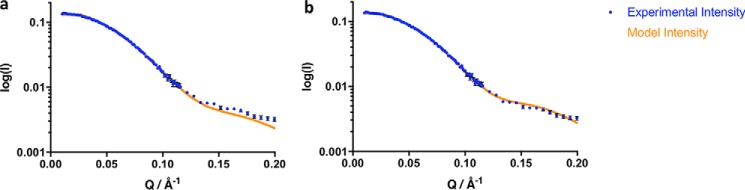
**Fits of the scattering curve of the oxidized state of the K75E/R78E/R108Q mutant of CPR to a two-state model.** The experimental data are shown as *points*, with *error bars* representing S.D., and the model fits are shown as *lines*. The wildtype crystal structure is used to represent the compact state, and either the model of Huang *et al.* (27) (*a*) or the structure of the ΔTGEE mutant (24) (*b*) is used to represent the extended state.

In view of the evidence that CPR exists in an equilibrium between a compact and a more extended conformation (9, 11, 22, 27), we analyzed the scattering data in terms of such an equilibrium using the program MultiFoXS (33). The compact state was represented by the crystal structure of soluble (N-terminally truncated) oxidized human CPR (Protein Data Bank code 3QE2 (12)). To represent the more extended state, we used either the model we described earlier (27), based on NMR and SAXS data on wildtype CPR, or alternatively molecule A in the crystal structure of the ΔTGEE mutant of CPR, which has a deletion in the flexible hinge and a more extended structure than the wildtype enzyme (Protein Data Bank code 3ES9 (24)). Analysis using either of these models for the extended state gave satisfactory fits (χ^2^ < 2) to the scattering curves (Fig. 3); the fitting parameters are given in Table 1. We also analyzed the data using a pool of 10,000 conformations generated by MultiFoXS by allowing the flexible hinge, and hence the FMN-binding domain, to move with respect to the FAD and linker domains; this did not give a significantly better fit to the data.

For the wildtype enzyme, the scattering curve is fit best by the compact crystal structure with only 10% extended structure. For the mutant, however, a good fit requires inclusion of a significantly greater proportion of an extended conformation. Either of the two models for the extended state can give a satisfactory fit, although, depending on the model chosen, the fraction of the extended conformation can vary from 16 to 33% (Table 1). We conclude, therefore, that the abolition in the mutant CPR of two salt bridges stabilizing the compact conformation (27) does indeed lead to an increase in the proportion of the extended, cyt *c*–reactive conformation in both the oxidized and reduced states and that this in turn leads to an increase in the amplitude of the burst phase of the reduction of cytochrome *c*.

### Structure of the complex between the CPR mutant and cytochrome c

The particular strength of SANS in studying macromolecular complexes arises from the ability to exploit solvent contrast variation by using buffers of different H_2_O/D_2_O composition (34, 35). This arises from the very different neutron scattering properties of hydrogen and deuterium so that H_2_O and D_2_O have very different scattering length densities. Hydrogen/deuterium exchange enables contrast matching and elimination of the scattering contribution of one component of a complex to specifically highlight the other component and therefore determine its structure and the relationship between them. Isotopically normal (hydrogenated) protein shows a “match point,” where it is rendered invisible in a SANS experiment, at ∼40% D_2_O. Perdeuterated protein cannot be “matched out” even in 100% D_2_O, but “match-out labeled” protein can be prepared by expressing the protein in *Escherichia coli* grown in 85% D_2_O (36). The precise match points of the hydrogenated cytochrome *c* and the match-out deuterated CPR mutant were measured to be 43 and 100% D_2_O, respectively (37) (Fig. S2).

Thus, when the complex between cyt *c* and the CPR mutant is prepared using isotopically normal cyt *c* and match-out deuterated CPR mutant, the scattering from the two components of the complex can be individually measured by carrying out the experiments in 100 and 43% D_2_O buffers, respectively. In 70% D_2_O, where the scattering length densities of the two proteins are equally different from that of the solvent, the scattering from the whole complex can be measured.

**Figure 4.**
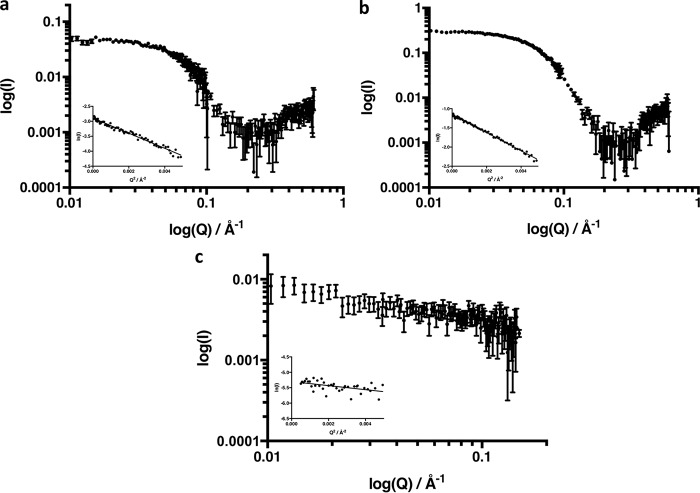
**Scattering curves of the complex between deuterated K75E/R78E/R108Q mutant CPR and cytochrome *c*.** *a*, complex fully visible (70% D_2_O). *b*, cytochrome *c* match-out (43% D_2_O). *c*, K75E/R78E/R108Q mutant CPR match-out (100% D_2_O). Guinier plots are shown in the i*nsets* in each case. Conditions were 100 mm BES, pH 7.0, at 15 °C with the appropriate percentage of D_2_O, as indicated in *parentheses*, to achieve the desired contrast.

**Figure 5.**
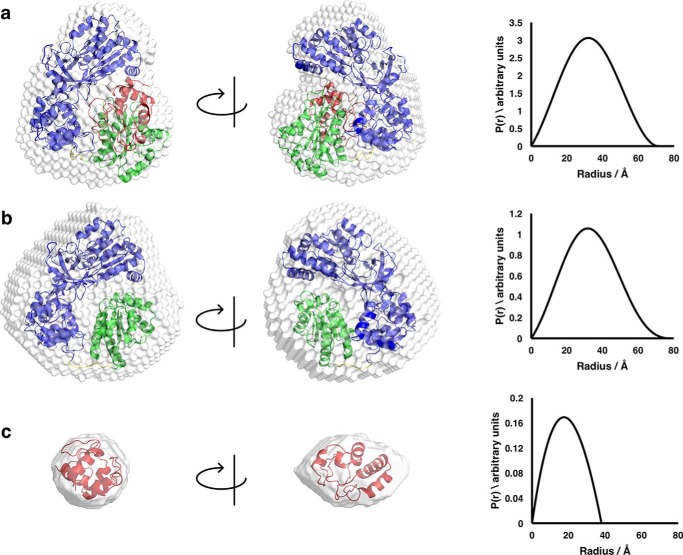
***Ab initio* scattering envelopes (*left*) and intraparticle distance distributions (*right*) of the CPR mutant–cyt *c* complex.** *a*, full complex. *b*, CPR mutant (cyt *c* match-out). *c*, cyt *c* (CPR match-out). In each case, the scattering envelope on the *right* is related to that on the *left* by a 90° rotation in the direction indicated by the *arrows*. In the structural models superimposed on the scattering envelopes, the FAD and linker domains of the CPR mutant are *blue*, the FMN domain is *green*, and cytochrome *c* is *red*.

The scattering curves from these three experiments are shown in Fig. 4, the derived hydrodynamic parameters are given in Table 2, and the scattering envelopes (calculated using DAMMIF) and the *P*(*r*) functions are shown in Fig. 5. When the full complex prepared in this way is studied in 70% D_2_O, the *R_g_* values are very similar to those for the CPR mutant alone, but the *D*_max_ value is significantly greater. Matching out the cyt *c* by carrying out the experiment in 43% D_2_O gives a *D*_max_ value the same as that obtained for the CPR mutant alone. Matching out the CPR mutant in 100% D_2_O gives the expected small values for *R_g_* and *D*_max_ of cyt *c*; although the signal-to-noise ratio for this poor scatterer is limited in this experiment, the *R_g_* and *D*_max_ are in satisfactory agreement with those measured for cyt *c* alone (Table 2). The *I*_(_*_Q_*_= 0)_ (intensity extrapolated to zero angle) values are as expected for the monomeric proteins at their respective concentrations.

**Table 2:** **Hydrodynamic parameters for CPR K75E/R78E/R108Q and its complex with cytochrome *c*** D-CPR, deuterated CPR; H-CPR, isotopically normal CPR.

Sample	*R_g_*		*D*_max_*^a^*	*I*_(*Q* = 0)_	Calculated molecular mass*^b^*
	Guinier	*P*(*r*)			
	Å		Å	*cm*^−*1*^	*kDa*
D-CPR K75E/R78E/R108Q mutant	25.2 ± 0.2	25.8	72	0.52 ± 0.0023	73 (69.6)
H-CPR K75E/R78E/R108Q mutant	25.0 ± 0.2	25.15	72	0.15 ± 0.00078	74 (69.6)
Full complex (70% D_2_O)	26.3 ± 0.8	26.9	77	0.05 ± 0.00082	81 (81.3)
Cyt *c* matched out (43% D_2_O)	26.0 ± 0.3	26.1	72	0.31 ± 0.0018	69 (69.6)
CPR mutant matched out (100% D_2_O)	11.5 ± 1.1	11.3	38	0.0051 ± 0.0002	12 (11.7)
Cyt *c* alone	12.2 ± 0.1	11.0	35	0.094 ± 0.0003	11 (11.7)

*^a^* All *D*_max_ values, determined from *P*(*r*) fits using GNOM in PRIMUS as part of the ATSAS suite, were rated as good (0.8) fits or better. All errors are <2 Å.

*^b^* Molecular mass values were calculated from experimental *I*_(*Q* = 0)_ values and protein concentrations estimated spectrophotometrically. Theoretical values from the amino acid sequences are given in parentheses.

Comparison of the bead-model *ab initio* envelopes derived from the scattering curves of the full complex and of the cyt *c* matched-out complex allows us to obtain an initial picture of the location of cyt *c* within the complex (Fig. 5). Superimposing atomic resolution models either from known crystal structures or from hypothetical models onto these envelopes permits visual confirmation of the fit of the data to the molecules in question. The model for the cyt *c* match-out experiment (Fig. 5*b*) was produced using the crystal structure of CPR (12) subjected to a random conformational search in which a pool of 10,000 conformational samples was created starting with the crystal structure and allowing the hinge residues linking the FMN-binding domain to the rest of CPR to be flexible. The model for the CPR match-out experiment (Fig. 5*c*), revealing only cyt *c*, was produced using the horse heart cyt *c* crystal structure (38).

To obtain a model for the full complex (Fig. 5*a*), we used the docking software HADDOCK together with the atomic-level information available on the interactions in the complex. An increase in ionic strength causes a substantial increase in the *K_m_* of CPR for cyt *c* (27, 39), indicating that electrostatic interactions are likely to be important in the formation of the complex. Lys^13^ of cyt *c* can be cross-linked to one of the carboxyl groups from two acidic clusters on the FMN-binding domain of CPR, Asp^207^-Asp^208^-Asp^209^ and Glu^213^-Glu^214^-Asp^215^ (40), and site-directed mutagenesis (41, 42) supports the involvement of the Glu^213^-Glu^214^-Asp^215^ cluster in the interface. Huang *et al.* (43) have recently used changes in NMR chemical shifts to study the interactions between the isolated FMN domain of rat CPR and cyt *c*, leading to two possible models for this complex. Utilizing the data from these studies in combination with our SANS data, we produced a model for the complex using the HADDOCK web server (44). A large number of models were initially produced, and the model with the lowest docking energies (electrostatics, desolvation, van der Waals, and restraints) was chosen as the starting point for further rigid body modeling. This yielded a model where the heme cofactor in cyt *c* was within a distance (<4 Å) of the FMN that would readily allow intermolecular electron transfer. The low-resolution nature of SANS means that the limiting factor in constructing the model is the scattering data, but it is clear that a model incorporating both the CPR mutant and cyt *c* fits the data much better than one involving the CPR mutant alone.

**Figure 6.**
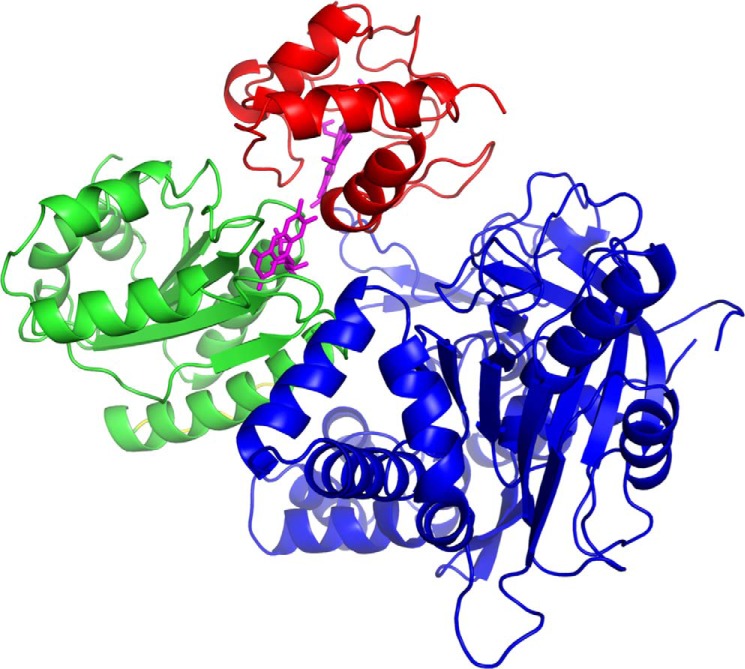
**The model of the CPR mutant–cyt *c* complex that best accounts for the data.** The FMN domain of the CPR mutant is shown in *green*, and its FAD and linker domains are in *blue*. cyt *c* is shown in *red*. The FMN of the CPR mutant and the heme of cyt *c* are shown in *magenta*.

To refine the model of the complex, the FMN domain and cyt *c* were fixed together to form a single rigid body, whereas the flexible hinge region connecting the FMN to the linker domain of CPR allowed the FMN domain/cyt *c* unit to move freely with respect to the rest of the CPR mutant molecule. We used a conformational search to produce a pool of 10,000 conformations, and theoretical SANS curves of each of these models were compared with the original data. It was found that a single model was able to fit the experimental data very well without the need to invoke a mixture of conformations. Five models, which were structurally indistinguishable, fit the data with χ^2^ < 2; the best of these, which had χ^2^ = 0.91, is shown in Fig. 6.

## Discussion

SANS lacks the resolution to be able to determine the orientation of cytochrome *c* and the domains of the CPR mutant in the complex in precise atomic detail. However, the model obtained in this work by combining global SANS information and atomic-level information (Fig. 6) clearly indicates a compact structure with extensive interdomain and intermolecular contacts, the latter involving residues in both the FMN and FAD domains of the CPR mutant.

This structure is generally similar to the reported crystal structure of the complex between the ΔTGEE mutant of rat CPR and rat heme oxygenase 1 (HO-1), another redox partner of CPR (45); it is interesting that two different mutants of CPR and two different redox partners give rather similar structures. However, there are clear differences between the two structures; in particular, the position of the FMN domain relative to the FAD domain is clearly different between the solution structure of the cyt *c* complex and the crystal structure of the HO-1 complex. This may be due to the different sizes of the redox partners in the two complexes (23 *versus* 12 kDa), the absence of NADP^+^ in the cyt *c* complex, or uncertainties in the 4.3-Å-resolution crystal structure in which the electron density for the FMN domain is clearly weaker than that of the FAD domain, particularly for one of the two molecules in the asymmetric unit (45). It is notable that the SAXS scattering curve of the ΔTGEE CPR–HO-1 complex is not identical to that calculated from the crystal structure (45), raising the possibility of differences between the solution and crystal structures.

In the model of the solution structure of the cyt *c* complex, there are several intermolecular polar side chain–side chain or side chain–backbone interactions between cyt *c* (residues Glu^21^, Gly^23^, Lys^25^, and Lys^27^) and residues Gly^267^, Arg^268^, Asp^280^, and Asn^359^ in the FAD domain of the CPR mutant, showing that the interprotein interface is not limited to the FMN domain of the reductase. The identified polar intermolecular interactions are listed in Table S1, although it must be noted that SANS does not allow us to define the interface with atomic-level precision. In this model of the complex, the conformation of the CPR mutant is somewhat more compact than in the free state, indicating that is “closing around” the cyt *c* in the complex. However, the conformation of the CPR mutant in the complex is still an extended one such that the isoalloxazine rings of the FAD and FMN cofactors are separated by ∼30 Å. In contrast, the distance between the FMN isoalloxazine ring and the heme of cyt *c* is <10 Å. This shows that the complex described here is one that is competent for interprotein electron transfer between the FMN and the heme of cyt *c* but not for intraprotein electron transfer between FAD and FMN, demonstrating that domain movement is required between the intramolecular and the intermolecular electron transfer steps in the CPR mechanism.

## Experimental procedures

NADPH and horse heart cytochrome *c* were purchased from Sigma-Aldrich. All other chemicals were of analytical grade.

### Protein expression and purification

The genes for human fibroblast CPR lacking the N-terminal membrane-anchoring region (46) (a kind gift from Professor C. R. Wolf, University of Dundee) and its K75E/R78E/R108Q mutant (27) were expressed in *E. coli* BL21 STAR (DE3) cells transformed with the pCS22 (cold-shock) plasmid vector carrying the CPR gene (21). Cells were grown to midlog phase (*A*_600_ of 0.6–0.8) in Terrific Broth medium containing ampicillin (100 μg ml^−1^) at 37 °C prior to induction by transferring the flasks to a prechilled incubator at 15 °C to exploit the cold-shock promoter (21). After 24 h at 15 °C, the cells were pelleted by centrifugation.

CPR was purified as described previously (21, 47) with modifications. The pellets were resuspended in a minimum of lysis buffer (100 mm Tris, pH 7.8, 100 μg ml^−1^ lysozyme) and sonicated on ice for 10 × 30 s. The suspension was centrifuged, and the supernatant was loaded directly onto a Q Sepharose ion-exchange column pre-equilibrated with wash buffer (100 mm Tris, pH 7.8). After washing with at least 2 column volumes of wash buffer, the protein was eluted with a gradient of 0–1 m NaCl in wash buffer. During elution, the absorbance was monitored at 280 and 450 nm; the fractions with the best 450 nm/280 nm ratio (presence of CPR confirmed by SDS-PAGE) were loaded directly onto a 2′,5′-ADP-Sepharose column pre-equilibrated with wash buffer. After a 2-column-volume wash, half a column volume of oxidation buffer (100 mm Tris, pH 7.8, 100 mm potassium ferricyanide) was washed over the bound protein to ensure that the flavins were in the oxidized state. After a further wash with wash buffer, the CPR was eluted with 20% glycerol in water, pH 7.0. The pure protein was eluted using a 20% glycerol solution rather than 2′-AMP to avoid undesired persistent binding of the 2′-AMP. A final stage of purification involved the use of size exclusion liquid chromatography (Superdex 200 Increase column) to isolate the purely monomeric form of the protein, essential in small-angle scattering experiments. The final eluent was buffer-exchanged into 100 mm Tris for refrigerator storage with the addition of 50% (w/v) glycerol for storage at −20 °C or into 100 mm BES, pH 7.0, for experimental work as required. An SDS-polyacrylamide gel of the purified protein is shown in Fig. S3. The protein concentration was calculated using a molar extinction coefficient ofϵ_450_ = 22,000 m^−1^ cm^−1^.

### Deuterated protein expression and purification

For preparation of deuterated protein, the gene for the CPR K75E/R78E/R108Q mutant lacking the N-terminal membrane-anchoring region was expressed in *E. coli* BL21 STAR (DE3) cells. The pCS22 vector–based construct was adapted for fermenter growth by switching the antibiotic resistance from ampicillin to kanamycin via a transposition reaction using the EZ-Tn5^TM^ <T7/KAN-2> Promoter Insertion kit (Epicenter). Cells were adapted for growth first from rich medium (LB) to hydrogenated minimal medium and then finally to 85% D_2_O–based deuterated minimal medium (36, 48). Plasmid-containing cells were grown to an *A*_600_ of ∼18 in a Labfors 2.3-liter Bioreactor (Infors, France) at 30 °C for 48 h in 85% deuterated minimal medium. Expression was induced in the fermenter by decreasing the temperature to 19 °C, and the expression was carried out for 22 h at the induction temperature. After centrifugation, the cell paste yield was 65 g from the 1.7-liter culture. CPR was purified as described above; the final yield of deuterated CPR from the total cell paste was 120 mg.

### Stopped-flow kinetics of cytochrome c reduction

Burst-phase kinetics of the reduction of cytochrome *c* by fully reduced CPR was studied under anaerobic conditions at 10 °C. The stopped-flow apparatus (Applied Photophysics, UK) was placed inside a glovebox (Belle Technologies, UK) in a nitrogen atmosphere with an oxygen content of 5 ppm or less. All solution kinetics studies were carried out in 100 mm BES, pH 7.0, buffer. A solution containing 10 μm CPR and 200 μm NADPH was incubated for 5 min in anaerobic conditions before starting any measurements. The reduced protein solution was rapidly mixed with an equal volume of 100 μm cytochrome *c* in the 2-μl flow cell, and the change in absorbance at 550 nm was recorded. 2000 data points were measured over a time period of 1 s to ensure that the burst phase and the transition to the slower steady-state phase were observed. To provide an initial reading for *A*_550_ in the absence of reduction, the cytochrome *c* solution was also mixed with the buffer solution only.

### SANS sample preparation

All solution neutron scattering studies were carried out in 100 mm BES, pH 7.0, at 10 °C. The appropriate ratio of D_2_O and H_2_O in the buffer was used for the desired contrast. CPR was mixed with a 5-fold molar excess of cytochrome *c* to induce complex formation. Either the samples were incubated on ice for 1 h before being measured immediately, or alternatively the complex was isolated using size exclusion chromatography (Superdex 200, GE Healthcare) at the SANS beamline before measurement. An SDS-polyacrylamide gel of the isolated complex is shown in Fig. S3.

### Solution scattering data collection

SANS measurements were carried out on D22, the high-flux neutron diffractometer at the Institut Laue-Langevin, Grenoble, France. Each protein sample of 2–5 mg/ml was measured in a 1-mm-path-length Suprasil quartz cuvette (Hellma) for a total of 1 h to gather data with a suitably high statistical precision. Data were recorded at two collimation lengths (5.6 and 2.8 m) and respective sample-to-detector distances (5.6 and 1.4 m) to provide a full range of momentum transfer *Q* from the Guinier region of the monomer to the solvent level of incoherent scattering. The two-dimensional ^3^He detector was positioned at different distances from the sample and off-centered in regard to the direct beam to provide a usable *Q* range of 0.001–0.5 Å^−1^ where *Q* = 4πsinθ/λ where 2θ is the scattering angle and λ is the wavelength (6 Å ± 10% in our measurements). The raw scattering data were reduced using the software GRASP^4^ (Institut Laue-Langevin), which included thickness and transmission scaling, empty cell and blocked beam subtractions, calibration to absolute intensity using incident flux measured at sample position, and azimuthal averaging, and then merged to produce the full scattering curves and buffer-subtracted and normalized for concentration as appropriate using National Institute for Science and Technology Centre for Neutron Research SANS reduction macros for IGORpro (50).

Contrast match points were estimated by determining and plotting the *I*_(_*_Q_*_= 0)_^1/2^ values of protein samples at various D_2_O/H_2_O ratios while maintaining a constant protein concentration (Fig. S2). The linear regression was used to estimate the D_2_O/H_2_O ratio at which the protein no longer contributed any coherent scattering (*i.e.* the *x* axis intercept). This estimate was then tested and confirmed before proceeding with contrast match SANS measurements on the protein complex.

### Data processing and modeling

Initial data processing and analysis were carried out using programs from the ATSAS suite (51). Determination of size parameters was performed using PRIMUS (52). *R_g_* was determined using the Guinier approximation and from *P*(*r*) plots; *D*_max_ and *P*(*r*) were calculated using GNOM (53). The PRIMUS Distance Distribution Wizard gave values of between 77 and 88% for the quality of *P*(*r*) for the different samples where values over 70% indicate a “good” solution. Model-independent *ab initio* molecular envelopes were generated using DAMMIF (54). Fifteen independent DAMMIF runs were averaged using DAMAVER (55) to obtain a typical molecular shape and filtered using DAMFILT to produce a refined model revealing only the most common structural features. High-resolution models were superimposed onto low-resolution dummy atom models using SUPALM (56) as part of the SASpy (57) plugin for PyMOL (58).

Rigid body modeling was carried out using software from the IMP (Integrative Modeling Platform) suite (59) and the ATSAS suite. A pool of 10,000 conformational samples was created using the RRT (rapidly exploring random tree) sampling tool, which was provided with a Protein Data Bank structure and identified flexible residues. These were the hinge residues linking the FMN-binding domain to the rest of CPR, specifically Gly^240^–Ile^245^, which are unresolved in the crystal structure (12); initial approximate positions for these residues were identified using the partial electron density from the original electron density map (12). In the model of the CPR–cyt *c* complex, the FMN-binding domain of CPR and cyt *c* were treated as a single rigid body so that the contact interface between them was maintained, and only this rigid body, connected to the rest of CPR by the flexible hinge, was allowed to move in the Monte-Carlo search. Theoretical scattering curves were calculated for each of the sampled conformations using CRYSON (49). A single best fit model to the experimental data was determined using MultiFoXS (33) in partial mode where precomputed scattering intensities were used.

An initial model for the CPR–cytochrome *c* complex was produced using the HADDOCK web server (44) by inputting predicted active interface residues on both the FMN domain and cytochrome *c* and allowing the program to automatically define peripheral residues. A large number of models were initially produced, and the model with the lowest docking energies (electrostatics, desolvation, van der Waals, and restraints) was chosen as the starting point for rigid body modeling using MultiFoXS (33) as described above.

## Author contributions

S. L. F., E. L. R., and G. C. K. R. conceptualization; S. L. F. and A. M. data curation; S. L. F., A. M., and G. C. K. R. software; S. L. F., A. M., and G. C. K. R. formal analysis; S. L. F., A. M., E. L. R., and G. C. K. R. supervision; S. L. F., E. L. R., and G. C. K. R. funding acquisition; S. L. F., A. M., J. D., J. B., and G. C. K. R. investigation; S. L. F., A. M., J. D., J. B., and G. C. K. R. methodology; S. L. F. and G. C. K. R. writing-original draft; S. L. F. and G. C. K. R. project administration; S. L. F., A. M., E. L. R., and G. C. K. R. writing-review and editing; A. M., J. D., and G. C. K. R. resources.

## Supplementary Material

Supporting Information

## Footnotes

4 C. Dewhurst, unpublished data.

## Footnotes

3 The abbreviations used are:

CPR: cytochrome P450 reductase
cyt *c*: cytochrome *c*
SANS: small-angle neutron scattering
SAXS: small-angle X-ray scattering
*R_g_*: radius of gyration
*D*_max_: maximum particle dimension
*P*(*r*): intraparticle pairwise distance distribution
HO-1: heme oxygenase 1
*I*_(_*_Q_*_= 0)_: intensity extrapolated to zero angle
BES: *N*,*N*-bis(2-hydroxyethyl)-2-aminoethanesulfonic acid.
