# Supplementary material for: Solution structure of the cytochrome P450 reductase–cytochrome c complex determined by neutron scattering
Source: J Biol Chem. 2018 Feb 23;293(14):5210–9. doi: 10.1074/jbc.RA118.001941 (PMC5892573; doi:10.1074/jbc.RA118.001941)
Supplement: Supporting Information [file supp_293_14_5210__index.html]

Solution structure of the cytochrome P450 reductase - cytochrome c complex determined by neutron scattering — Cytochrome P450 reductase - cytochrome c complex structure — Solution structure of the cytochrome P450 reductase–cytochrome c complex determined by neutron scattering — Cytochrome P450 reductase–cytochrome c complex structure — Supporting Information 

# Solution structure of the cytochrome P450 reductase–cytochrome *c* complex determined by neutron scattering

## Supporting Information

- Supporting Information - Three figures and one table of additional data
