# Supplementary material for: Solution structure of the cytochrome P450 reductase–cytochrome c complex determined by neutron scattering
Source: J Biol Chem. 2018 Feb 23;293(14):5210–9. doi: 10.1074/jbc.RA118.001941 (PMC5892573; doi:10.1074/jbc.RA118.001941)
Supplement: Supporting Information [file supp_RA118.001941_135356_1_supp_78600_p47807.pdf]

Supporting Material for

Solution structure of the cytochrome P450 reductase - cytochrome *c* complex determined by neutron scattering

Samuel L. Freeman, Anne Martel, Juliette Devos, Jaswir Basran, Emma L. Raven
and Gordon C.K. Roberts

Figure S1

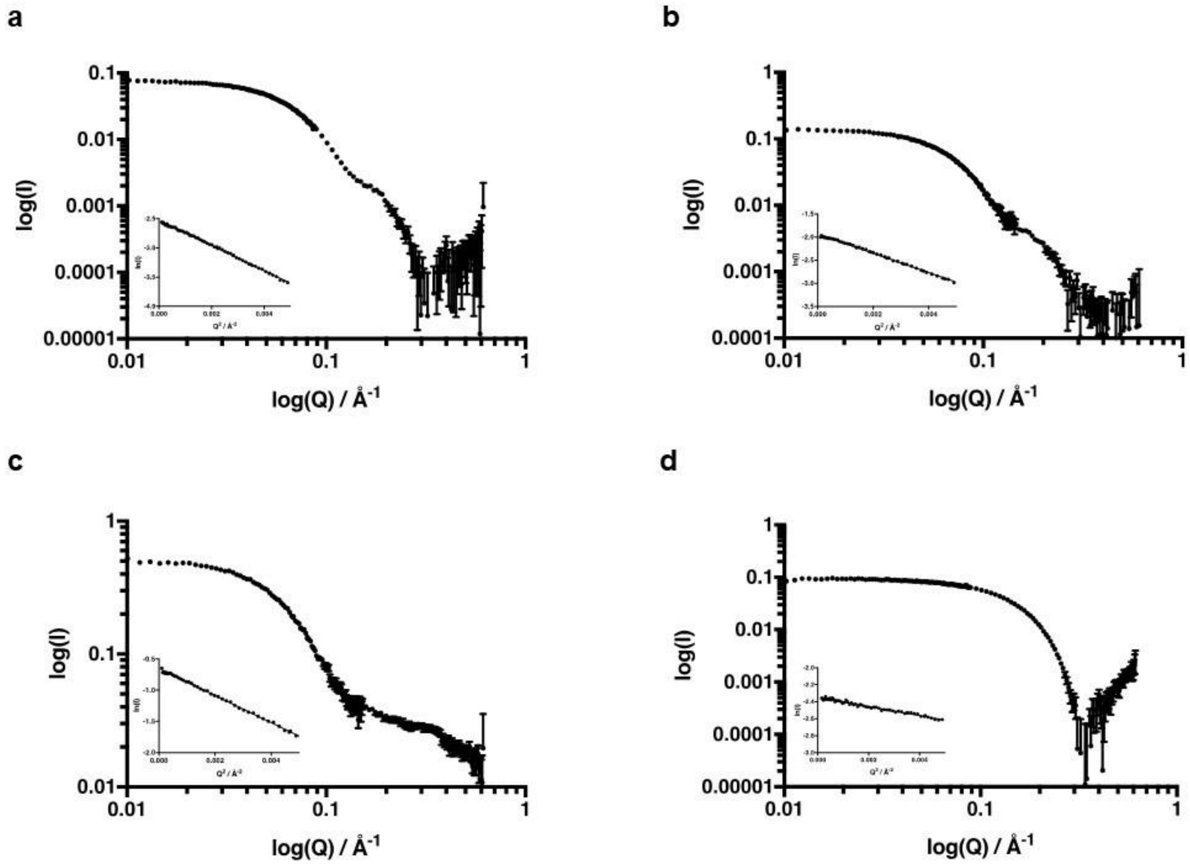

Scattering curves of individual proteins.
(**a**) wild-type CPR; (**b**) isotopically normal K75E/R78E/R108Q mutant CPR; (**c**) deuterated K75E/R78E/R108Q mutant CPR; (**d**) cytochrome *c*. Guinier plots are inset in each case. Conditions were 100mM BES, pH 7.0, made up with H_2O (**c**) or D_2O (**a**, **b**, **d**), at 15 °C.

Figure S2

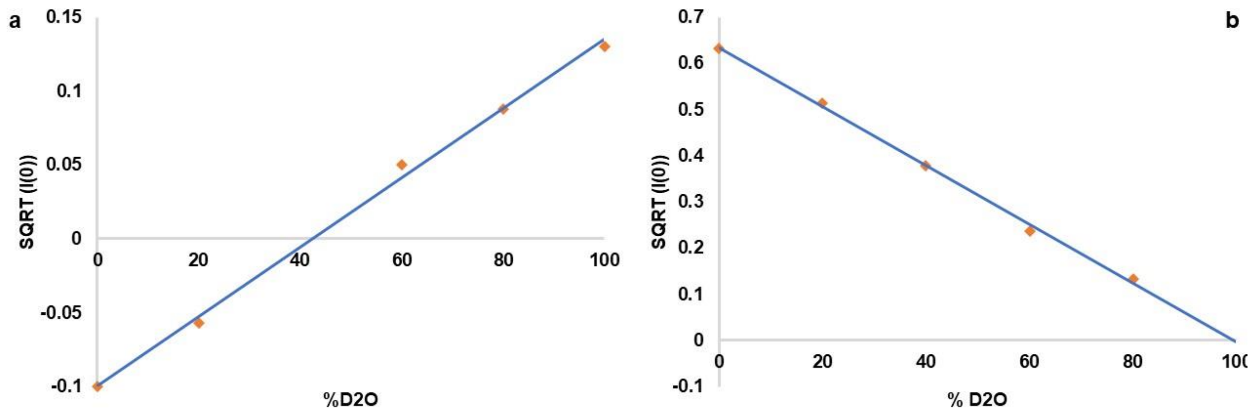

Contrast match point determination plots.
a, cytochrome *c*; **b**, deuterated K75E/R78E/R108Q mutant CPR

Figure S3

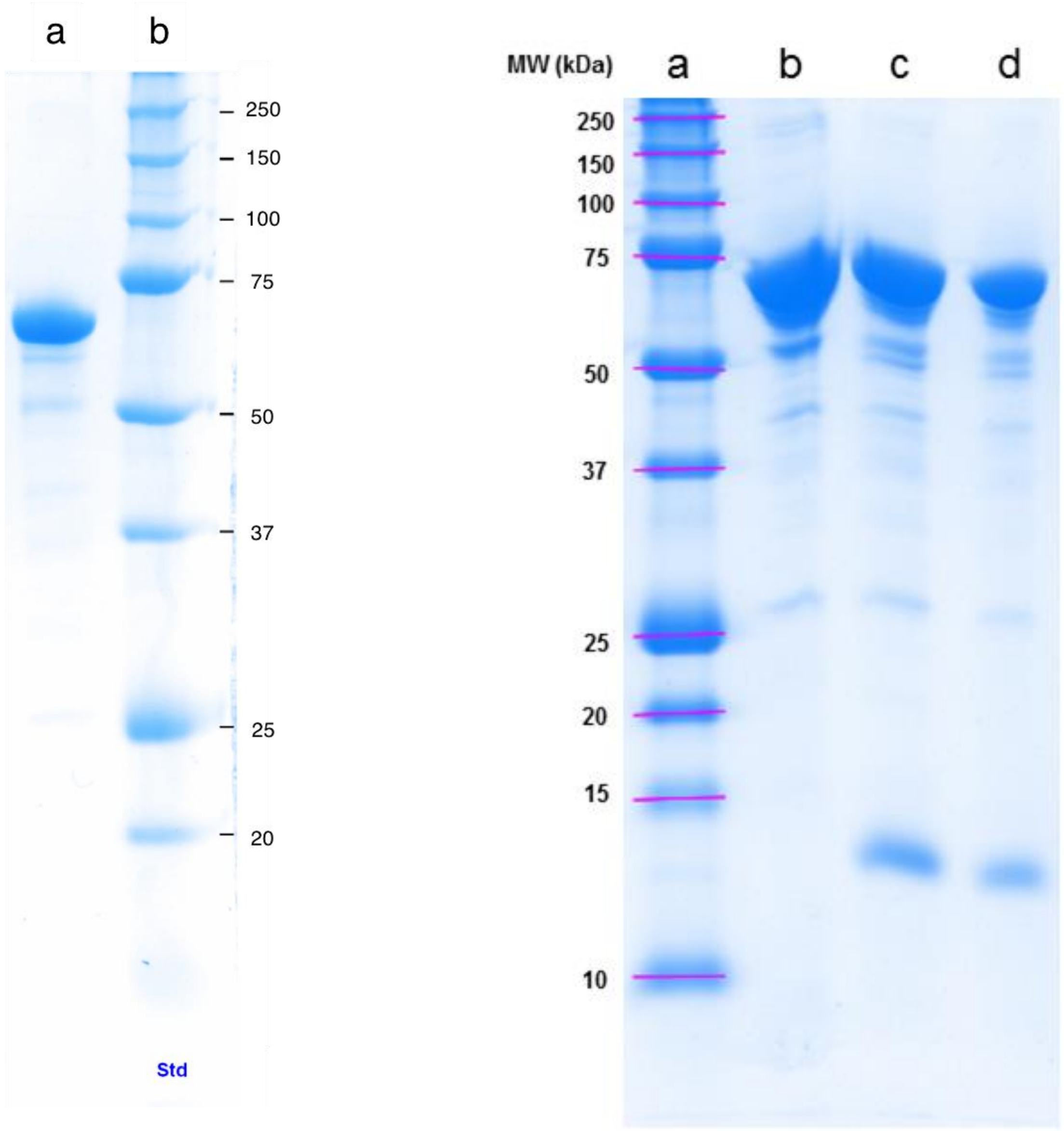

SDS-PAGE gels of the proteins used in this work. *Left*, purified CPR (lane **a**) and M_r markers (lane **b**). *Right*, the complex between deuterated K75E/R78E/R108Q mutant CPR and cytochrome c isolated by size-exclusion chromatography (lanes **c** and **d**), K75E/R78E/R108Q mutant CPR (lane **b**) and M_r markers (lane **a**); note that this gel was deliberately overloaded to reveal the very minor impurities.

Table S1

Polar intermolecular interactions in the complex between K75E/R78E/R108Q mutant CPR and cytochrome *c*

**CPR residue**	**Cyt *c* residue**
**FMN domain**[a]	
Gln 87	Ile 85
Glu 142	Gln 16
Asp 147	Lys 13
Glu 179	Lys 27
His 180	Gln16
**FAD domain**	
Gly 267	Lys 25
Arg 268	Gly 23
Asp 280	Glu 21
Asn 359	Lys 27 (CO)

[a] As discussed in the text, these residues were identified in studies of the interaction between the isolated FMN-binding domain of CPR and cyt *c* by Huang *et al*. (43) and incorporated into our model building.
